# The Future Role of Physician Associates in Ophthalmology Services

**DOI:** 10.22599/bioj.433

**Published:** 2025-06-04

**Authors:** Yu Jeat Chong, Matthew Azzopardi, Darren S. J. Ting

**Affiliations:** 1 Birmingham and Midland Eye Centre, Birmingham, UK; 2 Moorfields Eye Hospital, United Kingdom; 3 Academic Unit of Ophthalmology, Institute of Inflammation and Ageing, University of Birmingham, UK; 4 Academic Ophthalmology, School of Medicine, University of Nottingham, UK

**Keywords:** Physician associates, ophthalmology, workforce

## Abstract

As a response to increasing pressures on hospital eye services, the Royal College of Ophthalmologists (RCOphth) has started exploring the integration of physician associates (PAs) into ophthalmology as a means of expanding the ophthalmic workforce while maintaining high standards of care. However, the proposal has sparked a discussion within the ophthalmic community regarding the role of PAs in a specialty that already benefits from a well-established and specialized multidisciplinary team. Concerns have been raised about their short generalist training, which may not fully prepare them for the complexities of ophthalmic care, as well as the high cost of their integration compared to other healthcare professionals. Given these issues, upskilling existing allied health professionals and leveraging digital health innovations could be more effective solutions in addressing workforce shortages. The Royal College of Ophthalmologists (RCOphth), having endorsed the pilot scheme, bears the burden of proof to demonstrate the efficacy and value of PAs in this specialized field, ensuring that any workforce expansion aligns with the high standards expected in ophthalmic care.

In university debating, the term ‘framing the proposition’ refers to the way in which a proposition is set up and presented in order to shape the perspective from which a topic should be approached. One of the key aspects of a good proposition is to not only control the narrative but to also indicate the burden of proof to help win the debate.

Ophthalmology is recognized as the busiest outpatient-based specialty in secondary care and makes up 10% of the entire waiting list in the National Health Service (NHS England, 2023). The Royal College of Ophthalmologists (RCOphth) 2022 workforce census highlighted the significant strain NHS ophthalmic services are already under due to staff shortages, which is predicted to worsen without immediate action (The Royal College of Ophthalmologists, 2023b). In 2023, RCOphth initiated a 12-month pilot scheme to explore the feasibility of integrating physician associates (PAs) within ophthalmology as a means for workforce expansion (The Royal College of Ophthalmologists, 2023c). Their framing was that hospital eye services are facing increasing challenges to provide timely care to patients an issue that was further exacerbated by the recent COVID pandemic (Ting *et al*., 2020), the RCOphth is committed to promoting the highest standards of ophthalmic care. Following this, funds from NHS England were obtained to facilitate the exploration of the pilot PA framework in ophthalmology.

The proposed areas of work for a full-time PA (a 10-session, 5-day work week) include: two sessions of eye casualty, two sessions of scribe clinic, and one clinic/session each for telephone consult, preoperative assessment, intravitreal injection, ward work, and study/research. However, similar to the general medical field, there is considerable ambiguity and confusion surrounding the proposed role of PAs in the existing ophthalmic multidisciplinary workforce (Abbasi, 2024).

As noted by the RCOphth, “the aspects of clinical work that were previously the domain of the medically qualified ophthalmologist are now being delivered by a broader multidisciplinary team of qualified optometrists, orthoptists, ophthalmic nurses, ophthalmic clinical scientists, and technicians” (The Royal College of Ophthalmologists, 2023a). Other professions involved in the delivery of ophthalmic care include GPs and hospital doctors, electrophysiologists, visual function technicians, and orbital prosthetists. As members of the RCOphth, and working clinicians, we have a relatively clear idea of the training pathways and roles of our current multidisciplinary team (Table 1).

**Table 1:** Summary of Some Roles within the Multidisciplinary Ophthalmology Team.


JOB TITLE	JOB ROLE	QUALIFICATION PATHWAY	REGULATORY BODY

Ophthalmologists*	Medically and surgically trained specialist doctors with expertise in the diagnosis, treatment, management, and prevention of diseases of the eye and visual system.	Complete an undergraduate Bachelor of Medicine, Bachelor of Surgery (MBBS) degree (5–6 years), then train as a foundation doctor (2 years), then complete ophthalmology specialist training (7 years) to then undergo further sub-specialty training ([fellowship] not always; variable time frame) before becoming a consultant ophthalmologist.	General Medical Council (GMC)

**Optometrist**	Test vision, identify eye health problems, prescribe glasses, and fit contact lenses.	Complete a Bachelor of Science (BSc) in Optometry (3 years), then develop practical skills through the Scheme of Registration.	General Optical Council (GOC)

**Orthoptist**	Specialize in diagnosing and managing a range of eye conditions that largely affect eye movement and visual development.	Complete an undergraduate BSc in Orthoptics (3 years) or a postgraduate Orthoptics Master of Science (MSc) (2 years) after completing another undergraduate degree (preferably science-related) with Lower Second-Class honours	Health and Care Professions Council (HCPC)

**Ophthalmic Nurse**	Provide care to patients in a range of ophthalmic settings including eye casualty, pre-assessment, intravitreal injections, minor operations, and Botox injections Scrub nurses assist surgeons in theatre.	Complete a full-time nursing degree (3–4 years), longer if part time.	Nursing and Midwifery Council (NMC)

**Dispensing Opticians**	Give advice on vision care, and supply glasses and contact lenses.	Complete a full-time diploma (2 years) followed by working under supervision (minimum 1 year), or day release training course combined with working (3 years), or distance learning course including on-the-job training (3 years)	General Optical Council (GOC)

**Ophthalmic and Vision Science Practitioner**	Assess the structure and function of the eye, carry out a diverse range of tests to measure field of vision, perform imaging of the eye, and electrophysiological investigation of the eye and visual pathways.	No definitive degree requirements, require A levels of equivalent level-3 qualification.	Health and Care Professions Council (HCPC), as Clinical Scientists

**Orbital Prosthetists**	Work with the ocular prosthetics department and the UK National Artificial Eye Services to provide custom-made artificial eyes for patients.	No definitive degree requirements.	Health and Care Professions Council (HCPC)


Ophthalmologists include Consultants, Fellows, Trainees, and Specialty and Specialist (SAS) Doctors. Fellows are doctors who finish training and undergo further sub-specialist training before becoming a consultant. SAS doctors (specialist, associate specialist and specialty doctors) are experienced and senior doctors in permanent posts. They have at least four years of full-time postgraduate training, two of which have been in ophthalmology. Note: In recent years, new roles such as Enhanced Practitioners (EPs), and Advanced Practitioners in Ophthalmology (APs) have emerged, in which allied health professionals are trained further for roles in specific sub-specialties such as ophthalmology (this would typically require 3–5 years of clinical practice as a minimum, and a 2–3 year Master’s degree).

PAs are a relatively novel group of qualified professionals, and any new patient-facing professional group needs appropriate and clear regulation (Abbasi, 2024). At present, PAs are to be regulated by the General Medical Council (GMC) from December 2024, a move which has been vehemently opposed by the BMA and the UK Doctors’ Association (Abbasi, 2024). Apart from compounding patients’ confusion as to the difference between doctors and PAs, burdening the already heavily criticized GMC with the regulation of PAs seems premature. Given that the GMC does not regulate any health profession other than doctors, this decision seems to be solely driven by political expediency rather than sound judgement (Abbasi, 2024).

Becoming a fully qualified PA involves completing a three-year undergraduate degree in the UK, followed by a two-year postgraduate PA program. In most cases, the undergraduate degree has to be bioscience or health-related, though non-healthcare or non-biosciences Bachelor’s or integrated Master’s degrees with ‘relevant work experience’ may also be considered on an individual basis (Swansea University, 2023). The postgraduate PA training focuses principally on general adult medicine in hospital and general practice, rather than sub-specialty care (National Health Service, 2018). With time it is possible that PAs would be able to gradually learn and obtain a skill set that would help them work in sub-specialty settings. However, given that other members of the well-defined and specialized ophthalmic multidisciplinary team are already obtaining further sub-specialization and re-defining their clinical roles, one has to wonder what additional values this new PA role would contribute to ophthalmology. Furthermore, there is also the possibility that some PAs would have undergone an undergraduate or Master’s degree which is either loosely or not associated at all with healthcare. This would effectively mean that they would have undergone just two years of general clinical training before being deployed into clinical settings that often involve complex patients requiring extensive knowledge and experience.

Another issue to consider is the cost of integrating PAs into ophthalmology. Their average starting annual salary is £44,000 even without subspecialty experience in ophthalmology (National Careers Service, 2020d). This is considerably higher (by 20–37%) than the foundation doctors and allied health professionals, who typically earn £28,000–£35,000 per year (British Medical Association, 2024; National Careers Service, 2020b; National Careers Service, 2020c; National Careers Service, 2020a), and similar to the starting salary of first-year ophthalmology trainees (£43,923 per year), even though the latter group would have undertaken a significantly longer training pathway and have more clinical experience (i.e., 2 years of pre-clinical training, 3 years of undergraduate clinical training and 2 years of foundation training as a minimum) (British Medical Association, 2024).

While PAs might have a role in workforce expansion, they might be more suited for the general medical field rather than sub-specialty fields. To address the current ophthalmic workforce shortages more effectively (and cost-effectively), it would be better suited to focus on expanding the scope of work of allied health professionals, upskilling the existing workforce (such as through the RCOphth’s Ophthalmic Practitioner Training program, which trains postgraduate orthoptists, optometrists, ophthalmic nurses and other eye care practitioners in secondary care to further develop their skills in eye care), and introducing digital health innovations such as telemedicine and artificial intelligence technologies (Li *et al*., 2021; Ho *et al*., 2021; Bourlaki *et al*., 2024; In press).

We look up to the RCOphth for guidance, maintenance of professional standards, and quality. Given that NHS England has proposed the motion, and that the RCOphth has accepted the pilot, the burden of proof now lies on them to demonstrate the role and value of PAs in ophthalmology.

## Abbreviations

BMA, British Medical Association; BSc, Bachelor of Science; GMC, General Medical Council; MBBS, Bachelor of Medicine, Bachelor of Surgery; NHS, National Health Service; OPT, Ophthalmic Practitioner Programme; PAs, Physician Associates; RCOphth, Royal College of Ophthalmologists; SAS, Specialty and Specialist Doctors.
